# First case of Oropouche fever detected in the international border region of the Colombian Amazon: clinical characteristics and molecular diagnosis

**DOI:** 10.1590/0074-02760230221

**Published:** 2024-05-13

**Authors:** Juan Camilo Grisales-Nieto, Sérgio Luiz Bessa Luz, Valdinete Alves do Nascimento, Felipe Gomes Naveca, Luz Mila Murcia-Montaño, Kelly Natalia Romero-Vesga, Olga Eshter Bellido-Cuellar, José Joaquín Carvajal-Cortés

**Affiliations:** 1 Fundação Oswaldo Cruz-Fiocruz, Instituto Leônidas & Maria Deane, Núcleo de Patógenos, Reservatórios e Vetores na Amazônia, Manaus, AM, Brasil; 2 Fundação Oswaldo Cruz-Fiocruz, Instituto Leônidas & Maria Deane, Núcleo de Vigilância de Vírus Emergentes, Reemergentes ou Negligenciados, Manaus, AM, Brasil; 3 Amazonas Department Health Secretariat, Amazon Public Health Study Group, Leticia, Colombia; 4 Amazonas Department Health Secretariat, Public Health Surveillance and Epidemiology, Leticia, Colombia

**Keywords:** Oropouche, arboviruses, border, cross-border surveillance

## Abstract

**OBJECTIVES:**

We report the first case of Oropouche fever detected in the border region of Colombia.

**METHODS:**

Using a multiplex real-time polymerase chain reaction (PCR), genetic sequencing and clinical characteristics during the dengue epidemic in 2019, a total of 175 samples were analysed, from cases notified to the system epidemiological surveillance such as dengue.

**FINDINGS:**

The Oropouche virus (OROV) isolate from Leticia belongs to lineage 2 according to both M and S genome segments maximum likelihood (ML) analysis, shares a common ancestor with samples obtained in Esmeraldas, Ecuador and Turbaco, Colombia. The patient: a woman resident in the border neighbourhood of the municipality of Leticia had the following symptoms: fever, headache, retro-orbital pain and myalgias.

**MAIN CONCLUSION:**

This cross-border surveillance can be useful to give an alert about the entry or exit of arboviruses circulation in the region, which are often underreported in public health surveillance systems.

Oropouche fever is an arboviral disease caused by Oropouche virus (OROV), an arbovirus of the *Orthobunyavirus* genus in the *Peribunyaviridae* family (order *Bunyavirales*). It is transmitted to humans mainly by the biting midge of the *Culicoides* genus^1^ ^,^ ^2^ and potentially by *Culex quinquefasciatus* and some other species of the *Culex* genus.^2^ ^,^ ^3^ Since it was first isolated in Trinidad and Tobago in 1955, more than half a million cases and at least 30 major outbreaks of the disease in Central and South America have been reported. However, these data are difficult to quantify due to the lack of diagnosis and the similar symptoms with other arboviral diseases such as dengue (DENV), zika (ZIKV), chikungunya (CHIKV) and mayaro (MAYV).^3^ ^,^ ^4^ ^,^ ^5^

The OROV comprises three negative-sense single-stranded RNA segments, L (large), M (medium) and S (small). The small segment (S) (0.95 kb) encodes the nucleoprotein (N), the middle segment (M) (4.36 kb) encodes a polyprotein consisting of two glycoproteins (Gn, Gc) and a non-structural protein (NSm), while the large segment (L) (6.85 kb) encodes RNA-dependent polymerase RNA (RdRP).^2^ ^,^ ^3^ ^,^ ^4^ ^,^ ^6^

Brazil, Ecuador, Panama, Peru and Trinidad and Tobago have reported Oropouche fever in humans, with Brazil being the country with the highest number of cases of the disease in the region, mainly in the Amazonas, Acre, Bahia, Mato Grosso and Pará states.^1^ ^,^ ^2^ ^,^ ^4^

On the other hand, incidence values in humans have not been determined in some outbreaks. A relevant characteristic is related to a large number of infections reported in all the outbreaks described so far; the estimated incidence rates for OROV infection were determined through seroepidemiological surveys, in which groups of families were randomly selected.^4^

## MATERIALS AND METHODS

**Fig. 1:**
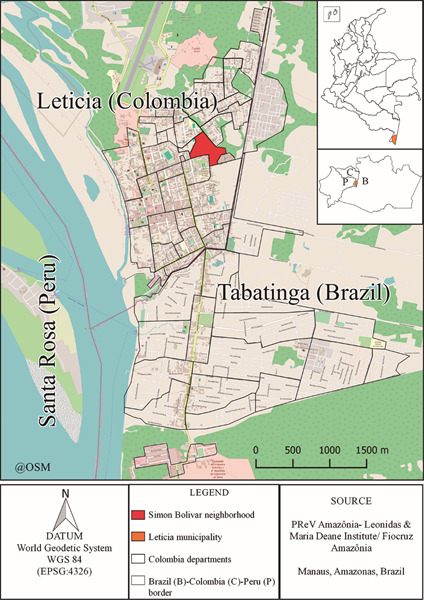
map of the Brazil-Colombia-Peru border and neighbourhood of the first case of Oropouche fever detected.

The twin cities of Leticia, Amazonas Department (Colombia) and Tabatinga, Amazonas State (Brazil), are located at the triple international border between Colombia, Brazil and Peru of the Amazon Region (Fig. 1). During the dengue epidemic in 2019 in Leticia, the counter samples were stored for the diagnosis of the disease which were taken in the first five days of symptoms and stored in ultra-freezers (-80ºC) in the public health laboratory of the Amazon Department (Colombia) - PHLAD. Subsequently, with the aim of knowing that other arboviruses, emerging and re-emerging viruses, circulate in the border region and in a cooperation agreement between the PHLAD and the Leônidas & Maria Deane Institute/ Fiocruz Amazonas (Brazil) - ILMD, the samples that had obtained results negative for the first differential diagnosis performed by the National Institute (INS) were analysed in November of 2020 at the ILMD. Samples for ZIKV, CHIKV, and DENV were analysed by real-time quantitative polymerase chain reaction with reverse transcription RT-qPCR, after a negative result, applied the protocol Taqman Fast Virus, through a multiplex trial of RT-qPCR is tested, analysing these samples for the MAYV and OROV,^7^ resulting in the first case of OROV in Leticia, of which molecular diagnosis and clinical description are made.

A total of 175 plasma samples with negative result for the first differential diagnosis for arboviruses were submitted to total RNA extraction by the Maxwell^®^ RSC, following the manufacturer’s instructions. After that, all RNAs were tested using a multiplexed reverse real-time PCR protocol for OROV (targeting the S segment) and MAYV (targeting the NSP1 coding region).^7^

Posteriorly, the OROV positive sample was submitted to a complementary DNA synthesis with the SuperScript IV Reverse Transcriptase and random primers (Thermo Fisher Scientific) according to manufacturer’s instructions. Subsequently, we performed conventional PCR targeting fragments S, M and L with previously published primers^2^ and Platinum SuperFi II Green PCR Master Mix (2X) (Thermo Fisher Scientific). For nucleotide sequencing, the PCR products were previously purified by PEG and sequencing reaction was carried out on an ABI3130 Genetic Analyser at the ILMD genomics platform.

*Ethics statement* - This study was developed within the surveillance activities of the public health laboratory of the Department of Amazonas (Colômbia) - PHLAD and is part of the activities contemplated in the cooperation work plan with the Leônidas & Maria Deane Institute/ Fiocruz Amazonas (Brazil) - ILMD, official letter no. 409/2019-GAB/ILMD/FIOCRUZ AMAZONAS of December 13, 2019, which refers to the joint work plan, for arbovirus surveillance in the border area between Brazil, Colombia and Peru, carried out in accordance with local legislation and institutional requirements.

## RESULTS

**Fig. 2:**
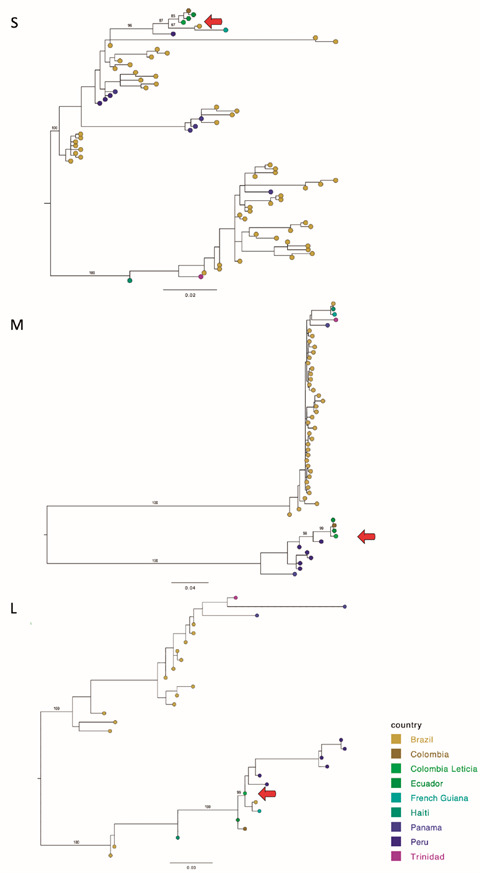
maximum likelihood (ML) analysis of the genome segments M and S, Oropouche virus (OROV) isolate from a patient in Leticia, Colombia.

According to both M and S genome segments maximum likelihood (ML) analysis, the OROV isolate from Leticia, Colombia shares a common ancestor with samples obtained in Esmeraldas, Ecuador and Turbaco, Colombia. Therefore, the OROV isolate from Leticia belongs to the M segment lineage 2, previously described by Gutierrez et al.^8^ Furthermore, our results for L segment analysis showed that the OROV isolate from Leticia also clusters with sequences from Ecuador and Colombia, but also with Brazil, Amazonas, French Guiana, and Peru (Fig. 2).

The dengue epidemic in 2019 was the largest epidemic recorded since 2011, when the first case of dengue fever was reported in Leticia. In 2019, Leticia notified 1,647 autochthonous dengue cases and 85 imported cases from municipalities in Brazil and Peru, presenting an incidence rate of 3,644.87 cases per 100,000 inhabitants. The female population was the most affected with 57.3% of the notified cases, 51.4% of the cases were between 5 and 29 years old and 19.6% in the indigenous population; in addition, Leticia there were three laboratory-confirmed ZIKV cases and nine CHIKV.

The patient was infected by OROV in August 2019, at the beginning of the dengue epidemic, with the following epidemiologic and clinic characteristics: a 63-year-old woman, resident in the Simón Bolívar border neighbourhood of the municipality of Leticia. This case was notified as DENV without warning signs in the Colombia National System of Public Health Surveillance in August, in the midst of the epidemic that occurred that year. The patient, declared to be a housewife, belonged to the indigenous *Cocama* community lowing and manifested intra-municipal displacement. The patient had the following symptoms: fever, headache, retro-orbital pain and myalgia.

## DISCUSSION

According to Sakkas et al.,^1^ OROV is an emerging disease that causes great concern in the regions of South America and Central America, it is classified as a neglected tropical disease and is considered that the prevalence, incidence rates in human populations, reservoirs and vectors have possibly been underestimated. Brazil, Peru, Panama and Trinidad & Tobago have reported the emergence and re-emergence of outbreaks of OROV, however most of these events have been reported in the Brazilian Amazon and probably have a silent transmission in other regions outside of the Amazon region, not detected by surveillance systems in public health.^1^ ^,^ ^9^ ^,^ ^10^

Human infections caused by OROV are characterised by being an acute febrile illness,^4^ similar to the infection caused by the DENV, its clinical evolution can last from two to seven days and is associated with a variety of symptoms, the most frequently reported during the major epidemics in Brazil, were: fever (100%), headache (79.3%), arthralgia (68.7%), myalgia (30%).^1^ ^,^ ^9^ ^,^ ^11^ ^,^ ^12^

Cities located in border areas have historically been considered highly vulnerable places, with political, economic and structural differences, that act as barriers to the control, diagnosis and treatment of diseases and access to health services.^13^ ^,^ ^14^ The twin cities of Leticia and Tabatinga are characterised by high population mobility, the deficiency of an aqueduct and sewage system, high flow of tourists, goods and services, as well as optimal ecological conditions to guarantee the presence of vector species incriminated in the transmission. This scenario may be different emerging and re-emerging diseases, such as Oropouche, into events with a high impact on public health at the triple international border between Colombia, Brazil and Peru.^14^ ^,^ ^15^

In Leticia in 2019, the first case of Oropouche found of the negative plasma samples processed for diagnosis of arboviruses, raises doubts the circulation of the other arboviruses simultaneously in lower incidence during outbreaks; for this reason, it is necessary to strengthen the cross-border surveillance, especially the molecular diagnosis of arbovirus by the public health laboratories of the twin cities of Leticia and Tabatinga.

Finally, it is necessary to understand the health-disease process in the international border areas, from the spatial and temporal dynamics of the disease and their vectors, the chain of transmission, and the epidemiological profile, for helping decision making in the local and regional level for the control of the different arboviruses in the cross-border areas.
